# Congenital diaphragmatic eventration and hernia sac compared to CDH with true defects: a retrospective cohort study

**DOI:** 10.1007/s00431-020-03576-w

**Published:** 2020-01-22

**Authors:** Kim Heiwegen, Arno FJ van Heijst, Horst Daniels-Scharbatke, Michelle CP van Peperstraten, Ivo de Blaauw, Sanne MBI Botden

**Affiliations:** 1 Department of Surgery, Division of Pediatric Surgery, Radboudumc-Amalia Children’s Hospital, Route 618, PO box 9101, 6500 Nijmegen, HB Netherlands; 2 Department of Neonatology, Radboudumc-Amalia Children’s Hospital, Nijmegen, Netherlands

**Keywords:** Congenital diaphragmatic hernia, Eventration, Hernia sac, Recurrence, Pulmonology

## Abstract

Congenital diaphragmatic eventration (CDE) and congenital diaphragmatic hernia (CDH) with or without hernia sac are three different types of congenital diaphragmatic malformations, which this study evaluates. All surgically treated patients with CDE or Bochdalek type CDH between 2000 and 2016 were included in this retrospective analysis. Demographics, CDH-characteristics, treatment, and clinical outcome were evaluated. In total, 200 patients were included. Patients with an eventration or hernia sac had no significant differences and were compared as patients without a true defect to patients with a true defect. The 1-year survival of patients with a true defect was significantly lower than patients with no true defect (76% versus 97%, *p* = 0.001). CDH with no true defect had significantly better short-term outcomes than CDH with true defect requiring patch repair. However, at 30 days, they more often required oxygen supplementation (46% versus 26%, *p* = 0.03) and had a higher recurrence rate (8% versus 0%, *p* = 0.006) (three eventration and two hernia sac patients). *Conclusion*: Patients without a true defect seem to have a more similar clinical outcome than CDH patients with a true defect, with a better survival. However, the recurrence rate and duration of oxygen supplementation at 30 days are higher than CDH patients with a true defect.

**Table:** 

**What is Known:** *• Congenital diaphragmatic hernia with or without hernia sac and congenital diaphragmatic eventration (incomplete muscularization) are often treated similarly.* *• Patients with hernia sac and eventration are thought to have a relatively good outcome, but exact numbers are not described.*	
**What is New:** *• Congenital diaphragmatic eventration and patients with hernia sac seem to have a more similar clinical outcome than Bochdalek type CDH with a true defect.* *• Patients without a true defect (eventration or hernia sac) have a high recurrence rate.*

**What is Known:**

*• Congenital diaphragmatic hernia with or without hernia sac and congenital diaphragmatic eventration (incomplete muscularization) are often treated similarly.*

*• Patients with hernia sac and eventration are thought to have a relatively good outcome, but exact numbers are not described.*

**What is New:**

*• Congenital diaphragmatic eventration and patients with hernia sac seem to have a more similar clinical outcome than Bochdalek type CDH with a true defect.*

*• Patients without a true defect (eventration or hernia sac) have a high recurrence rate.*

## Introduction

The clinical presentation of a congenital diaphragmatic hernia (CDH) can be variable, and the prognosis depends on multiple factors, with survival rates ranging from 70 to 80% [1, 2]. The most common type of a diaphragmatic defect is a posterolateral defect also known as Bochdalek hernia, which accounts for about 85% of the cases [3]. Bochdalek hernias occur due to an abnormal pleura-peritoneal fold development, where normally these folds fuse between the 5th and 7th week of gestation [4]. In this type of CDH, organs may herniate into the thorax because of the incomplete diaphragm. Congenital diaphragmatic eventration (CDE) is clinically seen and often treated as another subtype of CDH, although in the worldwide used CDH study group (CDHSG) scoring system, a specific classification for eventration, does not exist [3, 5, 6]. In CDE, there is incomplete muscularization of the (hemi)diaphragm, which results in a weakened muscularized dysfunctional diaphragm causing protrusion of abdominal contents into the thoracic cavity [4]. Clinical manifestations are diverse, varying from asymptomatic to life-threatening respiratory distress. Overall, it is thought to have a relatively good outcome, but exact numbers are not described [7], because most studies do not specify this subtype as a specific entity [2, 8–12].

CDH type Bochdalek patients can either have a true defect (“classic”) or a hernia sac, which has been described in 14–20% of cases. The latter type is often considered as a common variation of the “classic” CDH [3, 13–15]. In patients with a hernia sac, a non-muscularized pleuroperitoneal sac covers the herniated organs in the thorax [13, 14]. As however no explanation for the development of a hernia sac has yet been found, the question raises whether these patients with no “classic” true defect are more similar to the other patients with no true defect: CDE patients. Although the three different types described (CDH with a defect, CDH with a hernia sac and congenital diaphragmatic eventration) seem to be part of one spectrum, they may have been caused by different embryological events. The aim of this study is to evaluate whether there is a difference in clinical outcome between Bochdalek CDH with a true defect and patients without a true defect (hernia sac and eventration patients). A subanalysis will be performed to evaluate whether the presence of a hernia sac has more comparable clinical outcomes to CDE rather than Bochdalek type CDH with a true defect.

## Methods

### Inclusion and exclusion

**Fig. 1:**
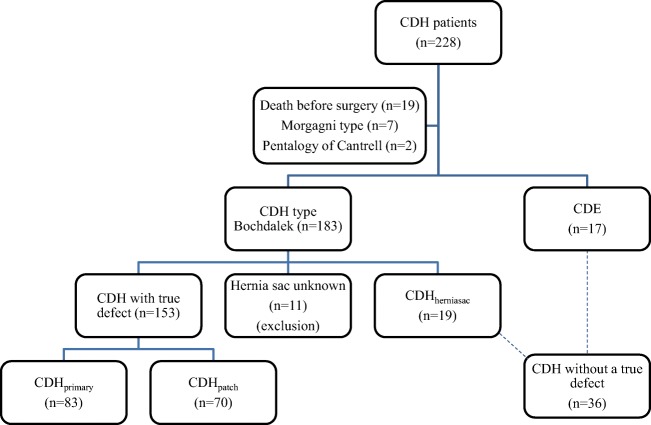
Exclusion and division of subgroups of the total group of CDH patients

All surgically repaired patients with a CDH diagnosed between January 2000 and December 2016 in a high volume center (Radboudumc-Amalia Children’s hospital, Nijmegen, the Netherlands) were included in this study. Exclusion criteria were incomplete data (no information on type of defect and presence of hernia sac), Morgagni hernias, and Pentalogy of Cantrell (Fig. 1). Patients that died before surgical repair were excluded, because no information on the type of defect was available. For long-term pulmonary outcomes at 30 days or at discharge, patients who died before this date were excluded in the analyses.

### Subgroups

Patients with a recorded CDE were compared to the patients with a Bochdalek type CDH with a true defect (CDH_truedefect_) and those with a hernia sac (CDH_herniasac_), and if comparable, they were combined to form one group (CDH_notruedefect_). The CDH_truedefect_ group was divided in primary closure (CDH_prim_) or patch closure (CDH_patch_), as a surrogate for the defect size.

### Protocol

Patient records were evaluated retrospectively for multiple parameters, such as demographics, perinatal parameters, and CDH characteristics. Prenatal diagnosis was based on either the standard 20-weeks’ gestation obstetric ultrasound or a prenatal ultrasound at another time point. Cardiac malformations were classified as major when hemodynamic effects occurred, as stated by the CDH study group (CDHSG) [16]. Because the defect size according to the CDHSG scoring system was introduced during the studied period and these data were missing in the majority of cases, the use of a patch respectively primary repair was used as a surrogate for the defect size [6]. After 2010, the patient was managed according to the standard of care for CDH patients at present time, supplemented by standard protocol proposed by the CDH Euroconsortium consensus paper [17]. Pulmonary hypertension was scored as either diagnosed on ultrasound or medication required. ECMO was offered to patients with severe respiratory failure and pulmonary hypertension when standard therapy failed according to the CDH Euroconsortium consensus [17]. All patch repairs were performed using PTFE-material (Goretex®). In the occurrence of a hernia sac, it was standard to excise the sac, or in some cases, it was used as an onlay tissueflap on the defect when it was closed by a patch. CDE was preferably repaired using plication of the diaphragm, although additional patch repair was used on indication. During follow-up, patients were evaluated on possible symptoms of recurrences and checked with standard two-plain X-rays postoperative, before discharge, during the follow-up, and on indication. Recurrences were diagnosed on two-plain x-rays of the thorax, in which a significant bulging or high position of the diaphragm was considered a recurrence.

### Outcomes

Primary outcomes of this study were survival and recurrence rate in the first year. Secondary outcomes were medical and surgical treatment (such as ECMO, primary or patch repair) and perioperative surgical complications such as hemorrhage, chylothorax, and recurrence rate during follow-up. Other outcome parameters were the need for oxygen supplementation, CPAP, or intubation at 30 days of life (DOL) and need of oxygen suppletion at time of discharge to home or another hospital. Mechanical ventilation or oxygen supplementation was used as a surrogate for lung development or short-term pulmonary status.

### Statistics

Values are expressed as means with standard deviations (SD), median with range, or in absolute numbers with percentages, when appropriate. Differences between patient groups were compared using the independent Student’s t test or Mann-Whitney U test for continuous variables and the chi-square or Fisher’s exact test for categorical variables. A *p* value of < 0.05 was considered statistically significant. Data management and analyses were performed using IBM SPSS Statistics 22.

## Results

In the study period, 228 patients were admitted for CDH in this tertiary center. Patients before and after 2010 (after the CDH Euroconsortium paper, as stated in the methods [16]) were compared on demographics, treatment, and outcomes. Comparison showed that there were no significant differences between these parameters during these two periods. The inclusion of the patients and division in the subgroups are demonstrated in Fig. 1, leaving 200 patients eligible for this study. They had a median follow-up of 11 years (range: 1–18). Of the total group, 77% had a CDH Bochdalek with a true defect (CDH_truedefect_), 10% had a CDH Bochdalek with a hernia sac (CDH_herniasac_), and 9% was diagnosed with an eventration.

### Demographics and treatment of the three groups: Bochdalek type CDH with true defect, CDE, and hernia sac

Demographic characteristics of the three groups, CDH_truedefect_, CDE, and CDH_herniasac_ patients, were described (Table 1). Only side of defect was different between the three groups, with a higher rate of right sided defects in the hernia sac and eventration cohort (*p* = 0.008). Patients with eventration and hernia sac had a higher rate of birth defects (31% and 26%) than patients with a true defect (11%, *p* = 0.02). Other birth defects reported were dysmorphic characteristics in three CDE patients versus two CDH_herniasac_ patients, vertebral anomalies in two CDH_herniasac_ patients and urogenital abnormalities (such as hydronephrosis) in two CDE and two CDH_herniasac_, and a hypospadia in two CDE patients.

**Table 1:** Demographics and perinatal characteristics of CDH with true defect, CDE, and CDH_hernia sac_

	CDE (*n* = 17)	CDH_herniasac_ (*n* = 19)	CDH_truedefect_ (*n* = 153)	*P* value	*P* value (CDE/CDH_herniasac_)
Gender – female	9 (52.9)	10 (52.6)	52 (34)	0.11	0.99
Gestational age in weeks^1^, median (range)	38.1 (31.3–41.7)	38.3 (30.1–42.7)	38.3 (27.7–42.3)	0.85	0.91
Birth weight in grams^1^,	2793 (817)	2997 (859)	3004 (609)	0.55	0.52
Mean (SD)					
Liver up	9 (52.9)	9 (47.4)	46 (30)	0.06	0.74
Apgar score^3^, median (range)					
1 min	8 (1–10)	8 (3–9)	6 (0–9)	0.13	0.87
5 min	9 (3–10)	9 (7–10)	8 (1–10)	0.06	0.51
Side hernia				**0.008**	0.28
Left	10 (58.8)	14 (73.7)	131 (85.6)		
Right	5 (29.4)	5 (26.3)	20 (13.1)		
Bilateral	2 (11.8)	0 (0)	2 (1.3)		

Missing: ^1^4, ^3^5

*CDE* congenital diaphragmatic eventration; *CDH*_*hernia sac*_ congenital diaphragmatic hernia, Bochdalek type, with hernia sac; *CDH* congenital diaphragmatic hernia

### Outcomes of the three groups: Bochdalek type CDH with true defect, CDE and hernia sac

CDE and CDH_herniasac_ patients all survived the first 30 days, compared to 82% of the patients with a true defect (*p* = 0.03). This higher rate of survival was also significant after 1 year (Table 2). The recurrence rate after 30 days was higher in the CDE and CDH_herniasac_, as none of the patients with a true defect developed a recurrence (*p* < 0.001). This was also the case after 1 year (however not significant, *p* = 0.10) (Table 2). Respiratory support after 30 days was 53% for CDE, 33% for CDH_herniasac_, and 26% for CDH patients with true defect (*p* = 0.01). At discharge, this was 20% versus 17% versus 4%, respectively (*p* = 0.02). Oxygen suppletion was higher after 30 days in patients with eventration (53%) and hernia sac (39%) compared to patients with true defects (26%), however not significant (*p* = 0.06). This was similar at discharge (27% versus 22% versus 10%, *p* = 0.10).

**Table 2:** Treatment and outcomes of CDH with true defect, CDE, and CDH_hernia sac_

	CDE (*n* = 17)	CDH_herniasac_ (n = 19)	CDH_truedefect_ (*n* = 153)	P value	P value (CDE/CDH_herniasac_)
Treatment					
Medical					
Pulmonary hypertension^1^	7 (43.8)	7 (41.2)	75 (56)	0.37	0.88
Use of inhaled nitric oxide^2^	3 (20)	6 (31.6)	67 (45.3)	0.11	0.70
ECMO	3 (17.6)	2 (10.5)	59 (38.6)	**0.02**	0.65
Surgical					
Method of repair				**0.02**	0.65
Primary	14 (82.4)	17 (89.5)	83 (54.2)		
Patch	3 (19)	2 (10.5)	70 (45.8)		
Non-closure of fascia^1^	1 (6.7)	1 (5.6)	41 (27.7)	**0.03**	1.00
Outcome					
Survival					
30-days	17 (100)	19 (100)	126 (82.4)	**0.03**	–
1-year	16 (94.1)	19 (100)	116 (75.8)	**0.01**	0.47
Surgical complications	6 (35.3)	5 (26)	53 (34.9)	0.76	0.72
Hemorrhage^3^	2 (12.5)	1 (5.3)	16 (84.2)	0.73	0.58
Chylothorax^3^	1 (6.3)	2 (10.5)	30 (19.7)	0.28	1.00
Recurrence of hernia					
30 days	1 (5.9)	2 (10.5)	0 (0)	**<0.001**	1.00
1 year^3^	2 (11.8)	3 (16.7)	7 (4.7)	0.10	1.00

Missing: ^1^3, ^2^2, ^3^1

*CDE* congenital diaphragmatic eventration; *CDH*_*herniasac*_ congenital diaphragmatic hernia, Bochdalek type, with hernia sac; *CDH*_*truefefect*_ congenital diaphragmatic hernia, Bochdalek group, without hernia sac; *ECMO* extracorporeal membrane oxygenation; *NICU* neonatal intensive care unit

### CDH patients with no true defect: hernia sac versus eventration patients

Patients without a true defect (eventration and hernia sac) were compared to each other, which show that there are no statistical significant differences in demographics at all (Table 1). Moreover, there were no differences in treatment or short-term outcomes (Table 2). Use of ECMO was not significantly different and neither was total ECMO run time (CDH_truedefect_ median 8 (range 1–20), CDE patients 11 days (range 9–13), and 6 days (range 4–8) in CDH_herniasac_ patients) (*p* = 0.18). Based on our clinical hypothesis, CDE (*n* = 17) and CDH_herniasac_ (*n* = 19) patients were pooled as one (clinical) entity so these patients with no “true” defect (CDH_notruedefect_, *n* = 36), meaning no direct adjacency between thorax and abdomen could be compared to the 153 Bochdalek hernia patients with a true defect (CDH_truedefect_).

### CDH without a true defect versus CDH with a true defect

In Table 3, the combined group (CDH_notruedefect_) is compared to CDH type Bochdalek with a true defect (CDH_truedefect_). This table shows that the patients in the CDH_notruedefect_ group were more often female (53% versus 34%, *p* = 0.04) and had higher Apgar scores. They were more often diagnosed with bilateral and right-sided hernias and liver up, which was diagnosed on prenatal ultrasound or perioperatively. The patients had no significant difference in rate of prenatal diagnosis (60% versus 58%, *p* = 1.00). As Table 3 also shows, other birth defects besides the diaphragmatic anomaly were more common among the CDH_notruedefect_ patients (29%) versus CDH_truedefect_ patients (11%) (*p* = 0.01).

**Table 3:** Demographics and perinatal characteristics of CDH patients with and without a true defect

	CDH_notruedefect_ (*n* = 36)	CDH_truedefect_ (*n* = 153)	P value
Gender – female	19 (52.8)	52 (34)	**0.04**
Gestational age in weeks^1^, median (range)	38.3 (30.1–42.7)	38.3 (27.7–42.3)	0.64
Birth weight in grams^2^, mean (SD)	2911 (834)	3004 (609)	0.48
Liver up^4^	18 (50)	46 (30)	**0.02**
Apgar score^6^, median (range)			
1 min	8 (1–10)	6 (0–9)	**0.05**
5 min	9 (3–10)	8 (1–10)	**0.02**
Side hernia			**0.02**
Left	24 (66.7)	131 (85.6)	
Right	10 (27.8)	20 (13.1)	
Bilateral	2 (5.6)	2 (1.3)	
Major cardiac malformations^7^	0 (0)	3 (2)	1.00
Chromosomal anomalies^8^	4 (11.4)	9 (6)	0.28
Other birth defects^7^	10 (28.6)	16 (10.5)	**0.01**

Missing: ^1^27, ^2^23, ^3^13, ^4^1, ^5^8, ^6^33, ^7^2, ^8^5

*CDH*_*notruedefect*_ eventration and hernia sac patients; *CDH*_*truefefect*_ congenital diaphragmatic hernia, Bochdalek group, without hernia sac; *CDH* congenital diaphragmatic hernia

Although there was no significant difference in presence of pulmonary hypertension between CDH_notruedefect_ and CDH_truedefect_ patients, ECMO was used more frequently in CDH_truedefect_ patients (Table 4). As this table also shows, survival on DOL 30 and after 1 year was significantly better for CDH_notruedefect_ (only one eventration patient deceased). When focusing on the surgical treatment, the abdominal fascia of CDH_notruedefect_ patients was mainly closed primarily (94%), compared to 72% of CDH_truedefect_ patients (*p* = 0.02). Recurrences of the diaphragmatic defect or eventration occurred more often and earlier in CDH_notruedefect_, in which 8% was diagnosed with a recurrence within 30 days (one eventration and two hernia sac) compared to 0% in CDH_truedefect_, (*p* = 0.006). This difference remained significant after 1 year (14% versus 5%, *p* = 0.05, Table 4). Not all of these recurrences required surgical repair, because some patients did not show any symptoms.

**Table 4:** Treatment and surgical outcomes of CDH patients with and without a true defect

	CDE_notruedefect_ (*n* = 36)	CDH_truedefect_ (*n* = 153)	P value
Treatment			
Pulmonary hypertension^1^	14 (42.4)	75 (56)	0.16
Use of inhaled nitric oxide^2^	9 (26.5)	67 (45.3)	**0.04**
ECMO	5 (13.9)	59 (38.6)	**0.005**
Non-closure of fascia^3^	2 (6.1)	41 (27.7)	**0.08**
Abdominal patch	1 (2.8)	27 (17.6)	**0.02**
Outcome			
Survival			
30-days	36 (100)	126 (82.4)	**0.002**
1-year	35 (97.2)	116 (75.8)	**0.006**
Surgical complications^4^	11 (30.6)	53 (34.9)	0.62
Hemorrhage^5^	3 (8.6)	16 (10.5)	1.00
Chylothorax^5^	3 (8.6)	30 (19.7)	0.12
Recurrence of hernia			
30 days	3 (8.3)	0 (0)	**0.006**
1 year^6^	5 (14.3)	7 (4.7)	0.05

Missing: ^1^22, ^2^7, ^3^8, ^4^1, ^5^2, ^6^6,

*CDH*_*notruedefect*_ eventration and hernia sac patients; *CDH*_*truefefect*_ congenital diaphragmatic hernia, Bochdalek group, without hernia sac; *ECMO* extracorporeal membrane oxygenation; *NICU* neonatal intensive care unit

When evaluating pulmonary outcome, respiratory ventilation at day 30 was needed in 42% of CDH_notruedefect_ compared to 20% in CDH_truedefect_ (*p* = 0.009). At discharge from our tertiary referral center, there was a significantly higher need for respiratory support for CDH_notruedefect_ compared to CDH_truedefect_ (18% versus 4%, *p* = 0.01). When correcting for ECMO patients only (CDH_notruedefect_, *n* = 5, and CDH_truedefect_, *n* = 59), this was 100% versus 47% (p = 0.05), respectively, at 30 days and 40% versus 8% at discharge (*p* = 0.12). In 32% of the non-ECMO patients of the CDH_notruedefect_ group, respiratory ventilation was required at day 30 versus 11% of the non-ECMO patients in the CDH_truedefect_ (p = 0.01) and 14% versus 3% at discharge (*p* = 0.04). Oxygen supplementation was needed more often in the group of CDH_notruedefect_ patients at 30 days (46% versus 26%, *p* = 0.03). At discharge, the difference was 24% versus 10%, but this was not statistically significant (*p* = 0.07).

### CDH without a true defect versus CDH patients with a true defect divided on type of repair (primary versus patch)

Table 5 compares the CDH_notruedefect_ patients with CDH patients with a true defect divided on the type of repair, as a surrogate for the defect size as previously explained (see methods). Primary repaired patients with true defects are abbreviated as CDH_prim_ (*n* = 83) and patch repaired patients as CDH_patch_ (*n* = 70). As Table 5 shows, there were no significant differences between CDH_notruedefect_ and CDH_prim_ patients regarding medical and surgical treatment. When the CDH_notruedefect_ group was compared to the CDH_patch_ group (considered the largest defects), CDH_notruedefect_ patients required less inhaled nitric oxide and were treated less often with ECMO (Table 5).

**Table 5:** Treatment and outcomes of CDH_notruefect_ versus CDH_Bprim_ and CDH_notruedefect_ versus CDH_patch_

	CDH_notruedefect_ (*n* = 36)	CDH_prim_ (*n* = 83)	*P* value	CDH_patch_ (n = 70)	P value
Treatment					
Pulmonary hypertension	14 (42.2)^1^	24 (32.4)^2^	0.32	51 (85)^3^	**<0.001**
Use of inhaled nitric oxide	9 (26.5)^4^	21 (26.3)^1^	0.98	46 (68)^4^	**<0.001**
ECMO	5 (13.9)	12 (14.5)	0.94	47 (67)	**<0.001**
Non-closure of fascia	2 (6.1^1^	10 (12.3)^4^	0.50	31 (46)^1^	**<0.001**
Abdominal patch	1 (2.8)	3 (3.6)	1.00	24 (34)	**<0.001**
Outcome					
Survival					
1-year	35 (97.2)	79 (95.2)	1.00	37 (52.9)	**<0.001**
Surgical complications	11 (30.6)	17 (21)^5^	0.34	37 (52.9)	**0.03**
Hemorrhage	3 (8.6)^5^	3 (3.7)^5^	0.36	13 (18.6)	0.18
Chylothorax	3 (8.6)^5^	10 (12.2)^5^	0.75	20 (28.6)	**0.02**
Recurrence of hernia					
30 days	3 (8.3)	0 (0)	**0.03**	0 (0)	**0.04**
1 year	5 (14.3)^5^	1 (1.2)^5^	**0.009**	6 (9.1)^6^	0.51
Pulmonary state 30 days					
O_2_ supplement	15 (45.5)^1^	6 (8)^7^	**<0.001**	24 (60)^8^	0.22
Mechanical ventilator support	14 (42.4)^1^	5 (6.7)^7^	**<0.001**	18 (45)^8^	1.00
Pulmonary state discharge					
O_2_ supplement	8 (24.2)^1^	4 (5)^7^	**0.01**	8 (24.2)^9^	1.00
Mechanical ventilator support	6 (18.2)^1^	0 (0)^7^	**0.01**	4 (12.1)^9^	0.73

Missing: ^1^3, ^2^9, ^3^10, ^4^2, ^5^1, ^6^4, ^7^8, ^8^30, ^9^37

*CDH*_*notruedefect*_ eventration and hernia sac patients; *CDH*_*prim*_ congenital diaphragmatic hernia, Bochdalek group, without hernia sac, primary repair; *CDH*_*patch*_ congenital diaphragmatic hernia, Bochdalek group, without hernia sac, patch repair; *ECMO* extracorporeal membrane oxygenation; *NICU* neonatal intensive care unit

When looking at outcomes, the survival rate of the CDH_notruedefect_ patients was comparable to CDH_prim_ (*p* = 1.00) and significantly better than CDH_patch_ (*p* < 0.001). However, the recurrence rate after 30 days was significantly higher for CDH_notruedefect_ compared to both the primary and patch repaired patients. After 1 year, the recurrence rate in the CDH_notruedefect_ patients was significantly higher compared to the CDH_prim_ patients (14% versus 1%, *p* = 0.009) and comparable to CDH_patch_ patients (*p* = 0.51). Pulmonary state at 30 days was worse for the CDH_notruedefect_ group when compared to the primary repaired patients (Table 5), which was also true for the primarily repaired patients specifically. After 30 days, the primary repaired patients without a true defect required oxygen in 39% versus 8% of the primary repaired patients with a true defect (p < 0.001), and for mechanical ventilator support, this was 36% versus 7% (*p* < 0.001). At discharge, this difference for primary repaired patients of CDH_notruedefect_ versus CDH_truedefect_ was for oxygen supplementation 21% versus 4% (*p* = 0.01) and mechanical ventilator support 18% versus 0% (*p* = 0.001).

## Discussion

This study suggests that CDE and a Bochdalek type CDH with hernia sac could have a more comparable clinical characteristics and surgical or short-term pulmonary outcome than patients with a true defect. This could be explained if the hernia sac is considered as an early form of an eventration. Both an eventration and a hernia sac then occur later in the embryology than the complete true defect in the diaphragm [3, 4, 15, 18]. The pleura-peritoneal fold has been closed in both scenarios, either just as a weak hernia sac or with little muscularization in case of an eventration, and may have been caused by different embryological events.

Patients with eventration have a significant better survival than the Bochdalek type CDH with true defect, which has been previously postulated as well [7, 19–22]. This was also stated for CDH patients with a hernia sac [13, 14, 23]. Despite the better survival rates, the two major findings of this study were the clinically relevant high recurrence rate (14%) and significantly longer need of respiratory support in patients with an eventration or with a hernia sac compared to (surviving) Bochdalek type CDH patients with a true defect.

The recurrence rate of CDH_notruedefect_ within 1 year was higher than in previous statements, where no recurrences in CDE were reported [7, 19–22]. Then again, the total number of recurrences found in the Bochdalek type CDH in this cohort (5%) is relatively low in comparison to other studies, and the sample sizes of both eventration and hernia sac patients are relatively small [8, 24]. Previous studies suggested that the severity or defect size of CDH would be a prognostic value for recurrence rates [24–27], but these cohorts were not adjusted for CDE and CDH with hernia sacs. The current study shows that during follow-up of the surviving patients, patients with no true defects have a higher recurrence rate than patients with a true defect. Recurrence rates of CDH_notruedefect_ even remain higher than Bochdalek patients with a patch, being a surrogate for large defect sizes in classic CDH. The higher recurrence rate might be explained by the fact that the use of a diaphragm patch for repair in patients with eventration or hernia sac is low, suggesting an underestimation of the strength of the repair. The native diaphragm may have been too weak for a reliable primary closure. The non- or partially muscularized tissue is probably not as strong as normal diaphragm and stretches more easily in the long run, causing more recurrences or new eventration. A lower threshold to use of patch closure in patients and resection of the hernia sac or part of the eventration may prevent the recurrences in both CDH patients with hernia sac or eventration of the diaphragm. However, due to the retrospective nature of these studies, confounding for the manner of hernia sac repair was not possible, a question for which prospective trial would possibly be required.

Another remarkable finding was that the patients with no true defect had a higher need for respiratory support, oxygen supplementation, both at 30 days of life and at time of discharge. For the groups separately, this clinically relevant difference was similar at discharge, however, not significant probably due to the small number of patients in the hernia sac and eventration group. These patients in this study initially had a better outcome but improved clinically less extensive than expected at 30 days and at discharge or even deteriorated. Because the numbers of pulmonary hypertension, treatment with inhaled nitric oxide, and ECMO were relatively low, pulmonary hypertension did not seem to explain this worse outcome for eventration patients of CDH patients with a hernia sac. Other explanations may be the presence of a more pronounced or underestimated pulmonary hypoplasia, a lesser functional diaphragm than previously thought (with higher recurrences) or the higher presence of other birth defects, which was shown in this study. Whatever the cause, the poor pulmonary condition with higher need for respiratory support at 30 days and at discharge warrants close pulmonary follow-up in this group of patients. In a future prospective study on this subject, it would be interesting to look for diaphragm motility disorder during follow-up, not only to explain difference in clinical follow-up but also to correct the outcomes for type of repair and its consequence on the function of the diaphragm. This could give us new insights in the type of surgery, type of defect, and functional outcome on the requirement of more ventilation and oxygen. Two previous studies by Levesque et al. (2019) and Oliver et al. (2019) showed a lower oxygen dependency after 28 respectively 30 days for hernia sac patients [28, 29]. However, all of these studies (including this study) are retrospective single center studies; therefore differences in diagnostics and treatment could give a bias (e.g., a difference in ECMO rate). Moreover, there may also be differences in the comparison to the patient groups of both centers in the non-hernia sac group, as, for example, in this study, eventration patients were excluded from the non-hernia sac group. Despite the differences between the patient groups, the rate of oxygen supply for hernia sac patients in our study seems to be comparable to the study by Oliver et al. (2019) (39% versus 44%). For the long-term follow-up, pulmonary function does seem to improve, as was also shown in other studies [13, 14, 23]. Wu et al. (2015) proved that the symptoms of their 86 symptomatic eventration patients resolved after surgery so timely diagnosis and treatment are related to lower respiratory morbidity (such as respiratory chest infection and cough) in these patients [19].

## Limitations

Due to the retrospective nature, data collection was incomplete and may be less reliable. Examples were the lack of access to data from the prenatal period such as the observed lung-to-head ratio (O/E LHR). For example, patch repair had to be used as surrogate for the size of the defect. The pooling of the data in the combined CDH hernia sac group and eventration group for subanalysis of comparison between a true and non-true defect may hypothetically be right but may have given a bias as well, based on the relatively small sample sizes of especially hernia sac and eventration patients. Although no significant differences in characteristics and outcome of these patients were found, a larger prospective cohort needs to confirm that these groups can be seen as one and give definite conclusions of the data given in this study. Also, the fact that some of the patients did not survive, mainly in the true Bochdalek group, could give a bias in the long-term outcome; however this group was not the focus of this study.

## Conclusion

The recurrence rate for patients without a true congenital diaphragmatic hernia defect (hernia sac and eventration) seems to be higher than for patients with a true Bochdalek type congenital diaphragmatic hernia. The similarities in these patients without a true defect found in this study can be an indication that these defects are often underestimated. The short-term outcomes of these patients are comparable to patients with a small true diaphragmatic defect, while the long-term prognosis is more comparable to patients with a large defect. Further studies are needed to evaluate whether the surgical techniques (to use or patch or not) for these specific defects need to be adjusted to prevent recurrences and improve the pulmonary outcome at 30 days of age and at discharge.

## Abbreviations

CDE: congenital diaphragmatic eventration
CDH: congenital diaphragmatic hernia
CDH_prim_: congenital diaphragmatic hernia, Bochdalek type, without hernia sac, primary repair
CDH_patch_: congenital diaphragmatic hernia, Bochdalek type, without hernia sac, patch repair
CDH_notruedefect_: congenital diaphragmatic hernia with no true defect, total group (eventration and Bochdalek type with hernia sac)
CDH_truedefect_: congenital diaphragmatic hernia, Bochdalek type, without hernia sac
ECMO: extracorporeal membrane oxygenation
NICU: neonatal intensive care unit
